# Phase I trial of the combination of bortezomib and clofarabine in adults with refractory tumors

**DOI:** 10.1007/s00280-026-04868-y

**Published:** 2026-03-14

**Authors:** Jibran Ahmed, Andre DeSouza, Shivaani Kummar, Lawrence Rubinstein, Geraldine O’Sullivan-Coyne, Jeevan Govindharajulu, William Herrick, Kate Ferry-Galow, Li Li, Deborah F. Wilsker, Murielle Hogu, Richard Piekarz, Robert Meehan, Mohamad Adham Salkeni, Sarah Shin, Brandon Miller, Jennifer Zlott, Lamin Juwara, Karen Gray, Laura Kuhlmann, Apurva Srivastava, Ralph E. Parchment, James H. Doroshow, Naoko Takebe, Alice P. Chen

**Affiliations:** 1 Division of Cancer Treatment and Diagnosis, National Cancer Institute, Bethesda, MD 20892 USA; 2 Leidos Biomedical Research, Frederick National Laboratory for Cancer Research, Frederick, MD 21702 USA; 3 Cancer Therapeutics Evaluation Program, National Cancer Institute, Bethesda, MD 20892 USA; 4 Center for Cancer Research, National Cancer Institute, Bethesda, MD 20892 USA; 5 Present Address: Knight Cancer Institute, Oregon Health and Science University, Portland, OR USA; 6 Present Address: Stephenson Cancer Center, University of Oklahoma, Oklahoma City, OK USA; 7 31 Center Drive, Room 3A44, Bethesda, MD 20814 USA

**Keywords:** Relapsed solid tumor, Lymphomas, Myelodysplastic syndromes, Proteasome inhibitors, Purine nucleoside analogs

## Abstract

**Purpose:**

The proteasome inhibitor bortezomib and purine nucleoside analog clofarabine combination had greater than additive activity in the NCI-ALMANAC preclinical screen. We conducted a phase 1 trial (NCT02211755) to evaluate the combination’s safety and efficacy in patients.

**Experimental design:**

We administered bortezomib subcutaneously on days 1 and 4, and clofarabine intravenously on days 1–5 of each 21-day cycle. The primary objective was to establish the safety, tolerability, and maximum tolerated dose (MTD) of bortezomib and clofarabine in patients with refractory solid tumors, lymphomas, or MDS. The secondary objective was to determine the effects of the combination on biomarkers of cell death and DNA damage response (DDR) in tumor biopsies.

**Results:**

Of 28 patients enrolled, 11 had a best response of stable disease (median 5 cycles; range 2–10 cycles), including 5 patients (4 from the solid tumor cohort, 2 of which were at MTD) with stable disease for ≥ 6 cycles. The MTD for the solid tumor cohort was 1.3 mg/m^2^ bortezomib on days 1 and 4, and 1.5 mg/m^2^ clofarabine on days 1–5 of each cycle. The MDS cohort closed prior to MTD determination, due to low accrual. The most common study drug related adverse events were hematologic. Two out of 3 patients with evaluable biopsies had increased markers of cell death, and 1 patient also had increased DDR markers after treatment.

**Conclusion:**

The combination of bortezomib with clofarabine demonstrated limited antitumor effects possibly due to the inability to reach the efficacious doses achieved in preclinical models.

**Supplementary Information:**

The online version contains supplementary material available at 10.1007/s00280-026-04868-y.

## Introduction

New drugs or drug combinations able to successfully target metastatic disease and tumors unresponsive to standard treatment are urgently needed to improve the survival of patients with advanced malignancies. Few candidates or combination strategies have achieved clinical success due to several factors, including molecular target heterogeneity, overlapping toxicities, and limited tumor-selective drug-delivery [1]. Using an unbiased, “hypothesis-free” phenotypic screening approach, several combinations of anticancer drugs were assessed in human tumor xenografts of NCI-60 cell lines [2]. The screen revealed that the proteasome inhibitor bortezomib and purine nucleoside analogue clofarabine combination conferred antitumor activity advantage over single-agent treatment in several* in vitro* and* in vivo* solid tumor xenograft models, and the combination treatment was well tolerated in preclinical studies [2]. To further support the preclinical hypothesis and a clinical trial, the NCI-ALMANAC study also interrogated mechanisms of action, resistance, and toxicity for bortezomib, clofarabine, as well as for the combination [2].

Bortezomib is a selective and potent small molecule inhibitor that reversibly inhibits the β5-subunit (PSMB5), and to a lesser extent the β1-subunit, of the proteasome [3]. Bortezomib treatment disrupts the degradation of proteins involved in the regulation of cell cycle, DNA repair and transcription, angiogenesis, migration, and apoptosis [4–6]. As a result, bortezomib triggers cancer cell death via several mechanisms, including by blocking the degradation of IκBα, a negative regulator of the nuclear factor-κB pathway which is constitutively active in these cancer cells, where it promotes proliferation, survival, and protects against apoptosis [3, 7], or by inducing pro-apoptotic Bax accumulation [8]. Bortezomib has received FDA approval for the treatment of hematological malignancies including multiple myeloma [9, 10], and mantle cell lymphoma [11, 12]. Bortezomib has shown activity in relapsed/refractory acute myeloid leukemia [13], but has only modest efficacy in solid tumors [14]. Moreover, drug resistance can occur due to mutations or upregulations of proteasome subunits, or alterations of gene and protein expression involved in antiapoptotic pathways [3].

Clofarabine is a second-generation purine nucleoside analog developed to improve efficacy and to minimize toxicity of the deoxyadenosine analogues, fludarabine and cladribine [15]. Clofarabine disrupts DNA synthesis by inhibiting ribonucleotide reductase and DNA polymerases [16] and has proven clinical efficacy in lymphomas and acute leukemias [17–19]. Clofarabine is FDA approved for the treatment of pediatric patients with relapsed/refractory acute lymphoblastic leukemia after at least two prior regimens. Preclinical evidence revealed that purine nucleosides can enhance the cytotoxic activity of bortezomib by down-regulation of Mcl-1, an apoptosis inhibitor binding executioner proteins BAX/BAK, that is upregulated by bortezomib and by inducing caspase-dependent apoptosis [20]. Recently, clofarabine has also been shown to induce apoptosis, pyroptosis as well as immunogenic cell death in models of melanoma and lung cancer [18] highlighting how clofarabine can target multiple pathways to enhance tumor cell killing.

Based on the activity of the combination of bortezomib and clofarabine in preclinical models [2], we conducted a phase 1 trial in patients with refractory solid tumors, lymphomas, or myelodysplastic syndrome (MDS) to further evaluate the safety and MTD of this combination. Because both bortezomib and clofarabine have been shown to induce apoptosis in cancer cells [12, 18], and clofarabine is known to induce pyroptosis [18] and DNA damage response (DDR) signaling [21], we also investigated potential mechanisms driving tumor response or resistance of cancer cells to treatment in patient biopsies.

## Patients and methods

### Eligibility criteria

Patients (age ≥ 18 years) were eligible if they had histologically confirmed solid tumors, lymphomas, or MDS that had progressed on standard therapy or for which no standard treatment options existed. Prior therapy with either clofarabine or bortezomib was not allowed. Prior chemotherapy, radiation therapy, or biologic therapy must have been completed at least 3 weeks (or ≥ 5 half-lives, whichever was shorter) before enrollment.

An ECOG performance status of ≤ 2, and adequate organ and marrow function defined as absolute neutrophil count of ≥ 1,500/µL, platelets ≥ 100,000/µL, total bilirubin ≤ 1.5 X institutional upper limit of (ULN), aspartate aminotransferase/ and/or alanine aminotransferase ≤ 3 X institutional ULN (or ≤ 5 X institutional ULN for patients with liver metastases), and creatinine ≤ 1.5 X ULN , creatinine within normal institutional limits or creatine clearance ≥ 60 mL/min/1.73 m^2^ for patients with creatinine levels > 1.5 mg/dL were required. Patients with sensory/motor neuropathy ≥ grade 2, active central nervous system metastases, or prolonged QTc interval were excluded.

Additionally, patients on the dose-expansion cohort were required to have tumor amenable to biopsy and willingness to undergo tumor biopsies.

### Trial design

This was an open-label phase 1 trial with the primary objective of evaluating the combination of bortezomib with clofarabine safety, tolerability, and maximum tolerated dose (MTD) in patients with refractory solid tumors or lymphomas; the study was amended in March 2016 to include patients with MDS. The study followed a 3 + 3 design with dose escalation enrolling at least 1 patient (up to 3 total) from each cohort per dose level until dose-limiting toxicity (DLT) was observed during the first cycle of treatment, at which point up to 6 patients in the cohort that had toxicities were independently enrolled to determine the MTD. Hematologic DLTs for patients in the solid tumor cohort were defined as: grade 4 neutropenia, grade ≥ 3 neutropenia with grade ≥ 3 infection, or febrile neutropenia; grade ≥ 3 thrombocytopenia; grade > 3 anemia or a drop of hemoglobin of > 2 g/dL from baseline within one week; any degree of lymphopenia, or leukopenia in the absence of grade 4 neutropenia was not considered a DLT. Due to the hematologic abnormalities associated with MDS, grade 3/4 cytopenias were not considered dose-limiting in patients in this cohort. Determination of hematologic DLTs in patients enrolled on the MDS cohort was decided by the study PI. Grade ≥ 3 non-hematological toxicity (except alopecia) felt to be related to study medications was considered dose-limiting in both cohorts with the following exceptions: grade *≥* 3 QTc prolongation was considered dose limiting if it occurred despite cessation of any concomitant medications associated with prolonged QTc; grade 3 diarrhea, nausea and vomiting were only considered dose-limiting if they were refractory to treatment; rise in creatinine to grade 3, or grade 3 electrolyte abnormalities (grade > 2 hypokalemia or hyperkalemia) not corrected to grade < 1 within 48 h with supportive treatment were considered dose-limiting. All Grade 4 rises in creatinine were dose limiting. The MTD was the dose at which no more than 1 of 6 patients of that dose level experience DLT during the first cycle of treatment, and the dose below that at which at least 2 (of ≤ 6) patients experience a DLT because of the treatment. The drugs were purchased by the NCI Division of Cancer Treatment and Diagnosis (DCTD).

Once the MTD was determined, a secondary study objective was included to determine the effects of the combination on biomarkers of cell death (apoptosis and pyroptosis), and DNA damage in tumor tissue. To achieve this objective, an MTD dose-expansion cohort was added to accrue a further maximum of 33 patients for tumor biopsy collection. Patients on the dose-escalation and dose-expansion cohorts were jointly evaluated for toxicity and response.

Bortezomib was administered subcutaneously on days 1 (day 2 for the dose-expansion cohort, to accommodate the biopsy collection schedule) and 4 and clofarabine was administered intravenously days 1–5 of each 21-day cycle (dose level detailed in Table 1). Because MTD was to be defined separately for the solid tumor and MDS cohorts, dose escalation was scheduled to proceed separately once each cohort was expanded to enroll 6 patients for DLT assessment. In January 2021, the MDS cohort was closed due to low accrual rate (3 patients total).

**Table 1:** Dose escalation schedule

Dose level	Bortezomib	Clofarabine
1	0.8 mg/m^2^	1 mg/m^2^
2	1 mg/m^2^	1 mg/m^2^
3	1 mg/m^2^	1.5 mg/m^2^
4	1.3 mg/m^2^	1.5 mg/m^2^
5	1.3 mg/m^2^	2 mg/m^2^
6	1.3 mg/m^2^	3 mg/m^2^
7	1.5 mg/m^2^	3 mg/m^2^
8	1.5 mg/m^2^	4.5 mg/m^2^
9	1.5 mg/m^2^	7 mg/m^2^
10	1.5 mg/m^2^	10 mg/m^2^

Exploratory objectives included: investigating the mechanism of action of the combination by examining markers of DNA damage and apoptosis in circulating tumor cells before and after treatment; characterizing the changes in MDS residual disease burden that occur with bortezomib and clofarabine treatment; and examining genomic alterations in circulating tumor DNA (ctDNA) that may be associated with response or resistance to clofarabine-bortezomib combination treatment.

Written consent was obtained from all patients. Protocol design and conduct followed all applicable regulations, guidance, and local policies (ClinicalTrials.gov Identifier: NCT02211755).

### Statistical considerations

This study used a modified 3 + 3 design, with dose escalation relying on the principles behind the Accelerated Titration Design [22]. For the PD dose-expansion cohort tumor biopsies were collected at three timepoints as described in Supplementary Fig. 1. The accrual ceiling for the dose-expansion cohort was set to 33 patients and reflected: (1) prior experience indicating a 90% likelihood of evaluability for a single tumor biopsy timepoint and (2) data from similar NIH Clinical Center phase 1 trials with paired triplet-biopsy pharmacodynamic endpoints. Biopsy triplets of sufficient quality from 12 patients would yield 90% power to detect treatment-related changes in individual PD endpoints of interest, equivalent to 1.6 standard deviations associated with the normal between-patient baseline variation of the markers, at the 2-sided 0.01 significance level (to accommodate multiple endpoints, while maintaining a reasonable over-all type 1 error), for the early or late time point, respectively, by the t-test.

### Tumor pharmacodynamics

Tumor biopsies for evaluating molecular PD response to the combination, by analyzing biomarkers of cell death and DNA damage (secondary endpoints), were mandatory for patients in the dose-expansion cohort. Up to 5 tissue cores ≥ 18-gauge in diameter and ≥ 1 cm in length were collected at baseline and again on cycle 1 day 1 (2–4 h post-clofarabine), and cycle 2 day 4 or 5 (prior to drug administration that day). Samples were processed according to the NCI Division of Cancer Treatment and Diagnosis (DCTD) Standard Operating Procedures (SOPs) available at https://dctd.cancer.gov/drug-discovery-development/assays/pharmacodynamic-biomarker-assays. Briefly, tumor biopsies were flash-frozen within 2 min from the time of collection and stored ≥-80 °C prior to processing to preserve labile biomarkers (e.g., phosphorylation) according to DCTD SOP340507 [23, 24]. Prior to microscopy evaluation, biopsies were thawed under neutral buffered formalin followed by paraffin embedding, sectioning and H&E pathology evaluation to determine adequacy for PD analysis (DCTD SOP340550) [25, 26] and analyzed using a fit-for-purpose, quantitative DDR immunofluorescent panel of nuclear pS139-H2AX (γH2AX), pS343-NBS1 (pNBS1), and pS824-KAP1 (pKAP1) (DCTD SOPs: LHTP003.07.05, LHTP003.07.25-ADM-01, LHTP003.07.26) [27, 28]. Validated multiplex immunoassays were conducted to evaluate markers of apoptosis (DCTD SOP341401) [29] and cleavage of pyroptosis-specific gasdermins (DCTD SOP341210) [30].

### CTC analysis in patient blood specimens

Blood samples for circulating tumor cells (CTCs) were collected to examine markers of DDR and epithelial to mesenchymal transition (EMT) (exploratory objective) during the dose escalation phase in patients with solid tumors and in the dose-expansion cohort according to the schedule in Supplementary Figure S1.

Each blood specimen for CTC analysis (7.5 mL) was collected into Cell-Free DNA BCT RUO blood collection tubes (Streck) and shipped and processed according to DCTD SOP LHTP003.08.19 (https://dctd.cancer.gov/data-tools-biospecimens/biospecimens-biobanks/resources/sops/specimen-collection). Amnis-based imaging flow cytometry assays were employed to assess CTC samples for EMT [31, 32] and for DDR biomarkers γH2AX (pSer139) and pNBS1 (pSer343) [28] as previously reported. DDR markers were considered increased if the percentage of biomarker-positive CTCs increased by at least 3-fold (or from 0 to ≥ 2 biomarker-positive CTCs in specimens containing ≥ 6 CTCs each) in one or more post-treatment specimens relative to baseline.

The following antibodies were employed for the immunofluorescence CTC analysis in the current study: MUC1 (Alexa-Fluor 488-conjugate clone E29, Santa Cruz Biotechnology); Pan-Keratin (Pan-CK) (Alexa-Fluor 555–conjugate clone C-11, Cell Signaling); Vimentin (Alexa-Fluor 568–conjugate clone EPR3776, Abcam); CD45 (Brilliant Violet 510-conjugate clone HI30, Biolegend); γH2AX (pSer139) (Alexa-Fluor 647-conjugate clone JBW301, Millipore); pNBS1 (pSer343) (Alexa-Fluor 750-conjugate clone EP178, Abcam).

### Analysis of circulating tumor DNA (ctDNA) blood

Whole blood was collected in three 10-mL Streck tubes in the dose-expansion cohort according to the schedule in Supplementary Fig. 1. A quantitative evaluation of plasma ctDNA was carried out to assess longitudinal changes in ctDNA levels (as a percentage of total circulating, cell-free DNA) and to identify any tumor genomic alterations that may be associated with response to the bortezomib and clofarabine combination (exploratory objective).

## Results

### Demographics

Twenty-eight patients were enrolled at the National Institutes of Health Clinical Center (Table 2) between October 2014–July 2023, of which 4 were enrolled on the dose-expansion cohort; 25 (89.2%) patients were evaluable for response. The dose-expansion cohort was closed early due to low accrual.

**Table 2:** Patient characteristics

Characteristic	No. of patients (%) Total *n* = 28
Median ages, years (range)	62.3 (30.4–81.2)
Male	17 (61%)
Female	11 (39%)
**Race**	
Asian	1 (3.5%)
Black	10 (35.7%)
Unknown	1 (3.5%)
White	16 (57.1%)
**ECOG performance status**	
0	0 (0%)
1	26 (93%)
2	2 (7%)
**Prior therapies**	
Prior lines of systemic therapy, median (range)	4 (1–9)
No. patients who had prior radiation therapy	9
No. patients who had prior surgery	22
No. patients who had prior chemotherapy	28
**Tumor type**	
Colorectal cancer	10
Cholangiocarcinoma	2
Follicular Lymphoma	1
Leiomyosarcoma	1
Malignant Peripheral Nerve Sheath Tumor	1
Pancreatic Adenocarcinoma	6
Renal Cell Carcinoma	1
Sclerosing Epithelioid Fibrosarcoma	1
Small Bowel Carcinoma	1
Small Cell Lung Carcinoma	1
Myelodysplastic Syndrome (MDS)	3

ECOG = Eastern cooperative oncology group

The median number of prior lines of systemic chemotherapy was 4 (range 1–9). Nineteen patients had prior surgery, and 9 had received prior radiation therapy. Median age was 62 years; sex and race statistics are summarized in Table 2.

The most frequent malignancy was colorectal cancer (10/28 patients; 35.7%), followed by pancreatic ductal adenocarcinoma (6/28 patients; 21.4%), MDS (3/28 patients; 10.7%), and cholangiocarcinoma (2/28 patients; 7.1%) (Table 2). In January 2021, the MDS cohort was closed due to low accrual.

### Clinical safety and MTD determination

All patients were evaluable for toxicity. The most common drug-related grade 3/4 adverse events related to study drugs were hematologic and included lymphopenia (64% or 18/28 patients; 50% grade 3/4), white blood cell decrease (36% or 10/28 patients; 18% grade 3/4), anemia (29% or 8/28 patients; 18% grade 3/4), neutropenia (29% or 8/28 patients; 21% grade 3/4) and platelet count decrease (25% or 7/28 patients; 14% grade 3/4) (Table 3). The only patient with follicular lymphoma (005) experienced grade 4 lymphopenia attributed to study drugs on cycle 1; the patient withdrew from the study after 2 cycles due to disease progression. DLTs occurred in 4 patients. One patient (011) at dose level 3 (1 mg/m^2^ bortezomib, 1.5 mg/m^2^ clofarabine) experienced grade 3 anemia. One patient (022) at dose level 4 (1.3 mg/m^2^ bortezomib, 1.5 mg/m^2^clofarabine) experienced grade 4 neutropenia and grade 4 thrombocytopenia. Two patients at dose level 5 (1.3 mg/m^2^ bortezomib, 2 mg/m^2^ clofarabine) experienced DLTs, including one patient (021) who experienced grade 4 neutropenia, and another patient (020) experienced grade 4 neutropenia and thrombocytopenia. Based on these data, the MTD was determined for solid tumor at dose level 4 (bortezomib 1.3 mg/m^2^ days 1 and 4; clofarabine 1.5 mg/m^2^ on days 1–5; 21-day cycles) with neutropenia and thrombocytopenia as the DLTs.

**Table 3:** Adverse events by patient. Worst grade (≥ 2 adverse events) considered possibly, probably, or definitely related to research drugs over the course of the trial for all dose levels and both cohorts (*N* = 28 total patients)

Adverse event	Gr 2	Gr 3	Gr 4
**Hematologic**			
Anemia	3	4	1
Lymphopenia	4	4	10
Neutrophil count decreased	2	1	5
White blood cell decreased	5	2	3
Platelet count decreased	3	3	1
**Digestive/Hepatic**			
Alanine aminotransferase increased	1		
Alkaline phosphatase increased	1	1	
Aspartate aminotransferase increased	1		
Diarrhea	1	1	
Hypoalbuminemia	2		
Nausea	2		
Vomiting	1		
Weight loss	1		
**Infection**			
Febrile neutropenia		2	
Sepsis			1
**Cardiovascular**			
Hypertension	1		
Ventricular arrhythmia		1	
**Dermatologic**			
Rash maculo-papular	1		
Skin and subcutaneous tissue disorders (hypersensitivity to Bortezomib)	1		
Urticaria	1		
**Electrolytes**			
Hypophosphatemia	1	1	
**Other**			
Hematoma		1	
Urine output decreased		1	
Fatigue	3		
Peripheral sensory neuropathy	1		

### Clinical activity

**Fig. 1:**
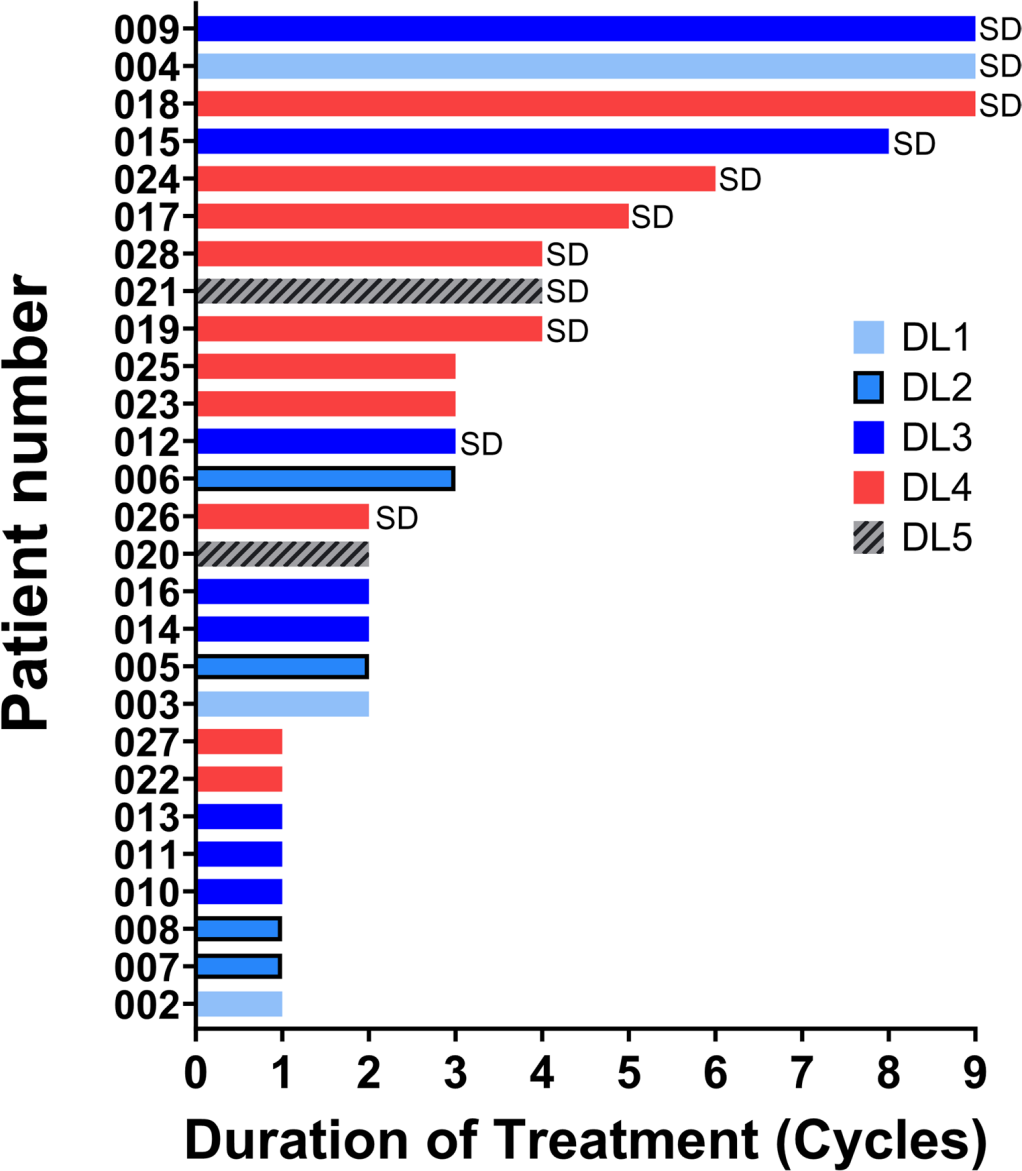
Clinical Activity. Duration of treatment (cycles) for patients on treatment depending on the Dose Level (DL) at time of enrolment. Patients who achieved a best response of Stable Disease (SD) are labeled on the bar plot

Patients on the dose-escalation and dose-expansion cohorts were jointly evaluated for response. The median number of treatment cycles was 2 (range 1–10) and median time of treatment was 1.5 months (range 0.2–8.2 months) (Fig. 1). Eleven of the 25 evaluable patients (44%) had a best response of stable disease (SD); the median duration of treatment for these patients was 5 cycles (range 2–10) (3.4 months, range 1.4–6.9 months). Five patients on different dose levels had durable SD for ≥ 6 cycles, including 2 patients with colon adenocarcinoma, 1 patient with cholangiocarcinoma, 1 patient with pancreatic cancer, and 1 patient with MDS. Two of the patients with durable SD were enrolled at the MTD level (1 pancreatic adenocarcinoma, and 1 colon adenocarcinoma). The length of SD was not correlated with the dose level.

Three patients were not evaluable for response. One patient (001) withdrew consent prior to starting treatment. Treatment was discontinued for one patient (010) prior to completion of the first cycle due to associated co-morbidities. One patient (013) was taken off treatment at the PI’s discretion, after completing one cycle of therapy, due to worsening ECOG performance status and safety concerns.

### Tumor pharmacodynamics

**Fig. 2:**
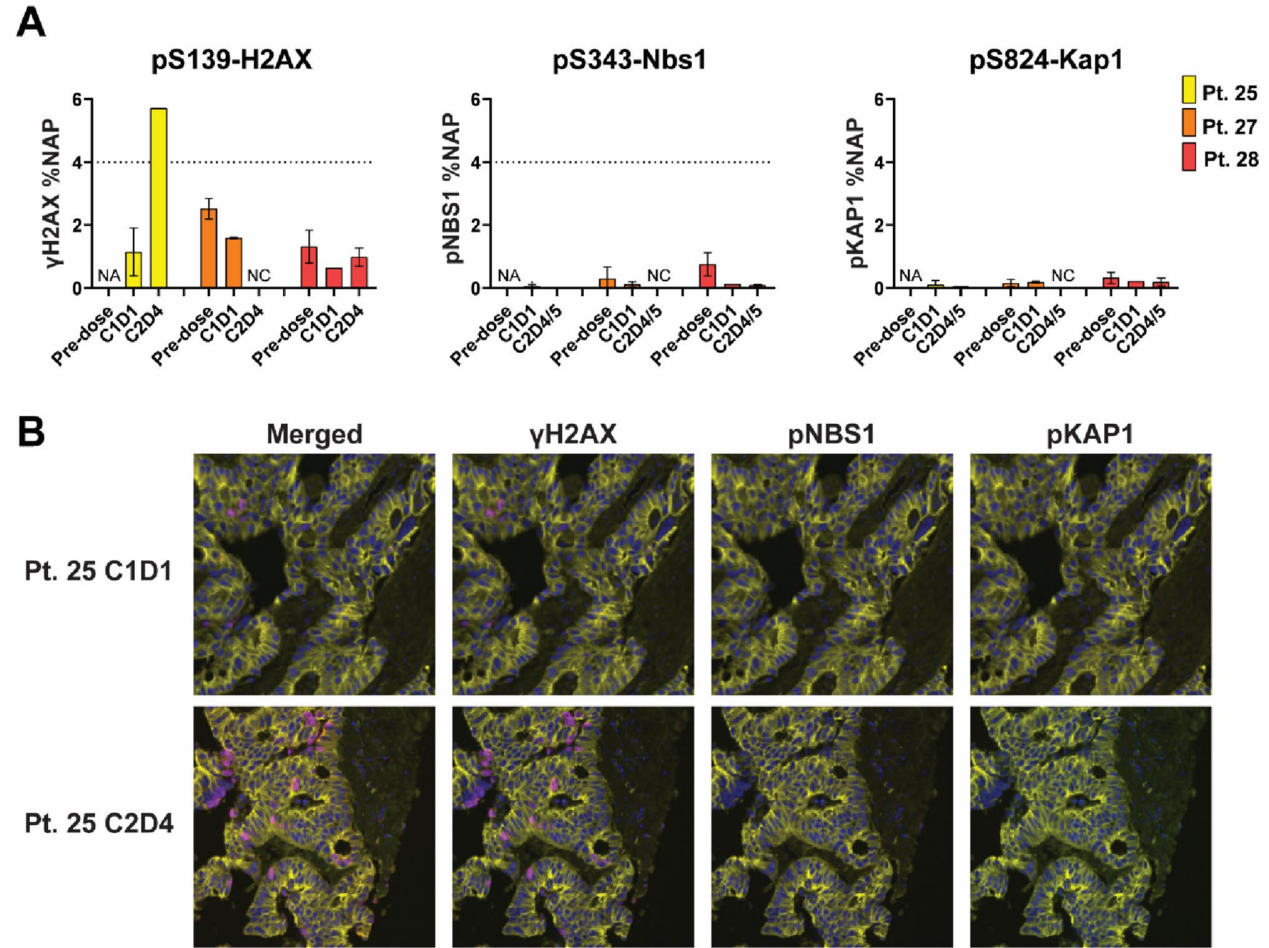
A. DNA Damage Response Markers in Treated Patients, quantitative immunofluorescent panel. B. DNA Damage Response (DDR) markers in patient biopsies, microscopy images. NA: not enough tumor for analysis; NC: not collected

Tumor biopsies were collected from 4 patients in the dose-expansion cohort of which 3 paired biopsies were evaluable for PD response (secondary objective). Treatment-induced DDR was evaluable in 3 patients at cycle 1 day 1, and in 2 patients at cycle 2 day 4 (Fig. 2A). DDR markers were not significantly induced at cycle 1 day 1 (2–4 h post clofarabine) compared to pre-dose levels in any of the 3 patients analyzed, but 1 patient (025) showed significant induction of γH2AX on cycle 2 day 4 (~ 72 h post bortezomib, 24 h post clofarabine) (Fig. 2A and B). Patient 025 was on DL4 but came off study after 3 cycles due to progressive disease.

**Fig. 3:**
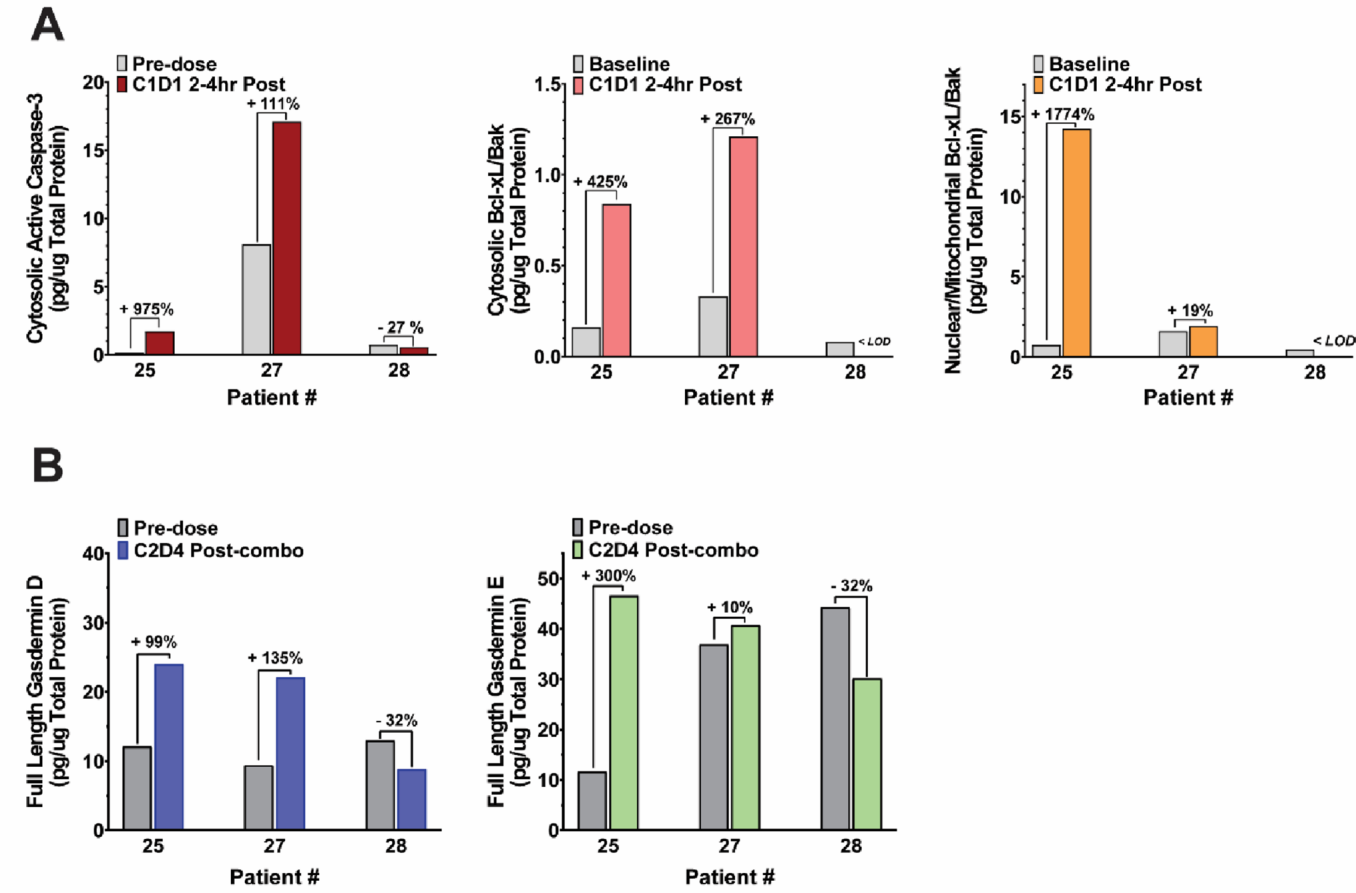
Cell Death Markers in Treated Patients.** (A)** Markers of apoptosis in clinical biopsies. **(B)** Markers of pyroptosis in clinical biopsies. < LOD: below limit of detection

For patients 025 and 027, increases in cleaved caspase-3 of over 100% were measured in the tumor samples after clofarabine alone at the cycle 1 day 1 2–4 h post-clofarabine timepoint compared to pre-dose levels (Fig. 3A), although the absolute level of cleaved caspase-3 in patient 025 remained low. This was accompanied by increases in tumor Bcl-xL–Bax heterodimer in cytosolic and nuclear/mitochondrial fractions of over 200% (Fig. 3A), indicative of resistance to apoptosis and consistent with high nuclear γH2AX foci and low cleaved caspase-3. Post-clofarabine levels of Bcl-xL–Bax heterodimer were below the assay limit of detection for patient 028.

Active caspase-3 is known to cleave and activate the pro-pyroptotic protein gasdermin E (GSDME) in response to chemotherapy drugs [33], and clofarabine treatment has been shown to induce GSDME-related pyroptosis in preclinical models of solid tumors [18]. Pre-dose and cycle 2 day 4 post-combination biopsy pairs were used to measure biomarkers of pyroptosis in response to combination treatment in all 3 patients. Increased levels of full-length gasdermin D (GSDMD) and gasdermin E (GSDME) were detected for patients 025 and 027 but not patient 028 (Fig. 3B). An increased level of the full-length gasdermins is inconsistent with the proteolysis associated with pyroptosis. However, direct measurement of cleaved GSDMD/E was not possible due to levels below the assay limit of detection from insufficient biopsy yields.

### CTC analysis in patient blood samples

CTCs isolated from 10 patients were analyzed using Amnis-based imaging flow cytometry assays to evaluate EMT [31, 32] and DDR biomarkers [28] (exploratory objective). Seven out of 10 patients (70%) had evaluable CTCs (≥ 6 CTC per 7.5 mL blood) at baseline. A decrease in total CTC numbers post treatment (at the latest point of collection) compared to cycle 1 day 1 was reported in 4/7 patients (57.1%) (Table 4). There was no correlation between CTC number changes and response to treatment or duration of SD.

**Table 4:** Changes in CTC numbers and pharmacodynamic biomarker levels in response to study drugs

Patient #	Cycles on treat.	Best Resp	# C1D1	# Final collection	% Vim C1D1	% Vim Final collection	% γH2AX C1D1	% γH2AX Post-dose*	% pNBS1 C1D1	% pNBS1 Post-dose*
**018**	9	SD	23	273	N/A	N/A	*0*	*38*	29	77
**019**	4	SD	340	77	23	33	0	9	25	5
**021**	4	SD	330	18	*0*	*100*	0	2	*4*	*33*
**023**	3	PD	107	43	0	0	0	0	3	8
**024**	6	SD	53	93	N/A	N/A	0	8	*0*	*75*
**027**	1	N/A	26	273	N/A	N/A	0	0	62	1
**028**	4	SD	33	10	N/A	N/A	20	14	70	86

C1D1 = cycle 1 day 1, N/A = not available, Vim = vimentin

Italic: biomarker values increased ≥ 3x after treatment, indicative of a DNA damage response

*Post-dose: highest value recorded after treatment initiation

Elevated baseline γH2AX and pNBS1 levels were detected in 1 patient, and 3 additional patients had elevated pNBS1 baseline levels (> 20%) (Table 4). Interestingly, 3 patients had increased DDR markers following treatment (Table 4): 1 patient (018) had increased γH2AX at cycle 5 day 1, and 2 patients had increased pNBS1 at cycle 3 day 1 (patient 019) and cycle 1 day 2 (patient 024). For 2 of these patients (018 and 024), the increase in DDR markers post treatment correlated with durable SD (9 and 6 cycles respectively) (Table 4).

Three patients had evaluable CTCs for EMT biomarkers, all of whom had a predominantly epithelial (Pan-Cytokeratin^+^ Vimentin^−^) phenotype at baseline. Hybrid (Pan-Cytokeratin^+^ Vimentin^+^) and mesenchymal (Pan-Cytokeratin^−^ Vimentin^+^) cells were detected in 2 patients post-treatment, but the increase in mesenchymal markers did not correlate with response (Table 4).

### ctDNA analysis in patient blood samples

Blood samples for ctDNA analysis (exploratory) were collected from a total of 7 patients. No responses were recorded to the study drugs and further ctDNA analysis was not performed.

## Discussion

In the preclinical NCI-ALMANAC study, that employed well characterized mouse xenograft models of human cancer, the combination of bortezomib and clofarabine had greater-than-additive growth inhibitory activity and modulated markers of apoptosis and DNA damage in responsive xenograft models [2]. These data served as the foundation for our phase 1 clinical study in patients with solid tumors and MDS. Importantly, the efficacious dose level of bortezomib in the combination regimen used by the preclinical NCI-ALMANAC studies (0.35 and 0.75 mg/kg [human equivalent dose 1.05 and 2.25 mg/m^2^], intraperitoneally every two days (Q2D) for 10 days) brackets the clinically approved dose of 1.3 mg/m^2^ administered intravenously on days 1 and 4 weekly [34–36]. However, the efficacious daily dose of clofarabine in the combination regimen used by the preclinical NCI-ALMANAC study (60 and 100 mg/kg [human equivalent dose 180 and 300 mg/m^2^], orally every two days (Q2D) for 10 days) is 3.5- and 5.8-fold higher, respectively, than the clinically approved daily dose of 52 mg/m^2^ in pediatric leukemia treatment [37]. In solid tumors, a single-agent clofarabine phase 1 trial established an MTD of 2 mg/m^2^ IV for 5 days, with a DLT of myelosuppression [38]. Our study established MTDs of the two agents in combination of 1.3 mg/m^2^ bortezomib and 1.5 mg/m^2^ clofarabine that are similar to previously reported single agent MTDs in solid tumors for bortezomib [14] and clofarabine [38], but this MTD of clofarabine in combination with bortezomib represents only 0.8% of the lowest tested dose that was efficacious in the preclinical study [2]. Achieving the efficacious preclinical doses of clofarabine in human patients with solid tumors may be limited, as indicated by the fact that patients with solid tumors developed neutropenia and required GCSF at clofarabine doses higher than 2 mg/m^2^ [38].

The main DLTs reported in our study, neutropenia and thrombocytopenia, are consistent with the known key side effects of bortezomib monotherapy, which additionally include peripheral neuropathy, and gastrointestinal toxicities [7]. Interestingly, the incidence of peripheral neuropathy in our study (only 1 patient reported grade 2 neuropathy) was lower than rates reported in multiple myeloma studies [7], possibly due to the lower dosage of bortezomib used in dose levels 1–3. Confounding factors in the assessment of toxicity include the heavily pre-treated nature of the patient population (median number of therapies 4, ranging 1–9) that can decrease bone marrow reserves, and increase the risk of myelosuppression caused by subsequent systemic therapy, which limited the exploration of higher dose levels for the clofarabine and bortezomib combination in our study.

Prolonged stable disease (≥ 6 months) was measured in 5 patients (Fig. 1). Tumor PD biomarker data suggest that the treatment induced DDR above the established baseline in at least one patient at the timepoints examined (Fig. 2A). These results suggest that the activity of this drug pair may be due to modulation of DNA damage as demonstrated by increased levels of γH2AX and pNBS1 and decreased levels of pHH3 in HCT-116 xenografts compared with vehicle and single-agent treatment [2].

In the NCI-ALMANAC study, the drug combination activated apoptosis in xenograft models, such as in the drug responsive HCT-116 model where treatment with the combination led to decreases in cytosolic and mitochondrial/nuclear survivin and mitochondrial/nuclear-associated lamin-B, and increases in caspase-3 activation and mitochondrial/nuclear-associated BAX, that were not measured in the non-responsive M14 xenograft model [2]. However, the caspase-3 activation reported in 2/3 patients on our study was accompanied by increases in tumor Bcl-xL–Bak heterodimer in cytosolic and nuclear/mitochondrial fractions (Fig. 3) indicative of intrinsic resistance to apoptosis [39]. Importantly, the absolute level of cleaved caspase-3 in patient 025 remained low, consistent with prior reports that co-localization of cytosolic cleaved caspase-3 and nuclear γH2AX foci is required to distinguish apoptosis from successful DNA repair and tumor cell survival [40]. Multiple mechanisms of resistance to bortezomib treatment have been described in literature, including mutations in the proteasome’s β5 binding pocket, increased basal level expression of PSMB5, increased expression of pro-survival and anti-apoptotic proteins such as heat shock proteins and IGF-1 receptor expression, and upregulation of pathways conferring resistance to apoptosis such as the Raf/MEK1, PI3-K/Akt pathways and cMET signaling [3, 12, 41].

Pyroptosis is a form of inflammatory cell death mediated by gasdermins which can overlap with other forms of cell death where caspases play important roles, including apoptosis [30]. Preclinical studies have shown that clofarabine treatment in melanoma and lung cancer cells increases levels of cleaved GSDME, a known pyroptosis mediator; furthermore clofarabine activates the non-canonical STING-NFκB pathway leading to increased expression of BAX, and ultimately induces both apoptosis and pyroptosis via the BAX/Caspase-3 pathway [18]. Increased levels of full length GSDMD/E were reported in 2 of our patients following treatment (Fig. 3B). Although the mechanism responsible for increased levels of GSDMD in our samples remains unclear, published reports suggest it may promote tumor progression by regulating anti-tumor immunity [42]. Clinical levels of cleaved GSDMD/E were either below the assay limit of detection, or paired-biopsies results were not available, precluding us from evaluating if pyroptosis occurred in the analyzed patients following treatment.

There was no correlation between changes in CTC numbers following treatment and response to therapy or duration of SD in the 7 patients with evaluable CTC numbers. Elevated baseline DDR levels, including elevated pNBS1, were detected in 4 patients (Table 4), indicative of genomic instability. Interestingly, for 2/3 patients, the increase in DDR markers post treatment correlated with durable SD (9 and 6 cycles respectively) (Table 4) and reflected increases in the same two DDR markers in the responsive preclinical model [2] .

Taken together, our PD data indicate that targeted drug-combination effects are detectable at the doses evaluated. However, due to low drug tolerability the combination did not offer clinical benefits in this heavily pretreated patient population.

## Supplementary Information

Below is the link to the electronic supplementary material.

Supplementary Fig. 1: Study Schema for The Phase 1 Clinical Trial. **(A)** Dose-escalation cohort: Bortezomib was administered subcutaneously on days 1 and 4 and clofarabine was administered intravenously days 1–5 of each 21-day cycle. Mandatory blood samples for circulating tumor cell (CTC) analysis were collected from patients with solid tumors only at baseline (pre-treatment), on cycle 1 day 2 prior to clofarabine administration and on day 1 of all subsequent cycles before drug administration. Optional blood samples for CTC analysis could also be collected at time of progression. **(B)** Dose-expansion cohort: Bortezomib was administered subcutaneously on days 2 and 4 and clofarabine was administered intravenously days 1–5 of each 21-day cycle. Mandatory biopsies were collected prior to clofarabine and bortezomib administration (up to 8 days prior to the start of treatment), on cycle 1 day 1 (2–4 h post-clofarabine), and cycle 2 day 4 or 5; an optional biopsy could be collected at time of disease progression. Blood samples for CTCs analysis were collected at baseline (pre-treatment); on cycle 1 day 1 (2–5 h after clofarabine administration); at the time of the biopsy on cycle 2 day 4 or day 5 (within ± 8 h of the biopsy); on days 1 and 4 of cycle 3 and all subsequent cycles before drug administration; and at time of disease progression. Blood samples for circulating tumor DNA (ctDNA) analysis were collected at baseline (pre-treatment); at the time of the cycle 2 (day 4 or 5) biopsy; on day 1 of cycle 3 and all subsequent cycles; and at time of disease progression. Full arrows indicate mandatory planned sample collections; dotted arrows indicate optional sample collections

## Data Availability

The original data presented in the study are included in the article/supplementary material. For further inquiries contact the corresponding authors.
