# Supplementary figures and images for: Phase I trial of the combination of bortezomib and clofarabine in adults with refractory tumors
Source: Cancer Chemother Pharmacol. 2026 Mar 14;96(1):25. doi: 10.1007/s00280-026-04868-y (PMC12987827; doi:10.1007/s00280-026-04868-y)

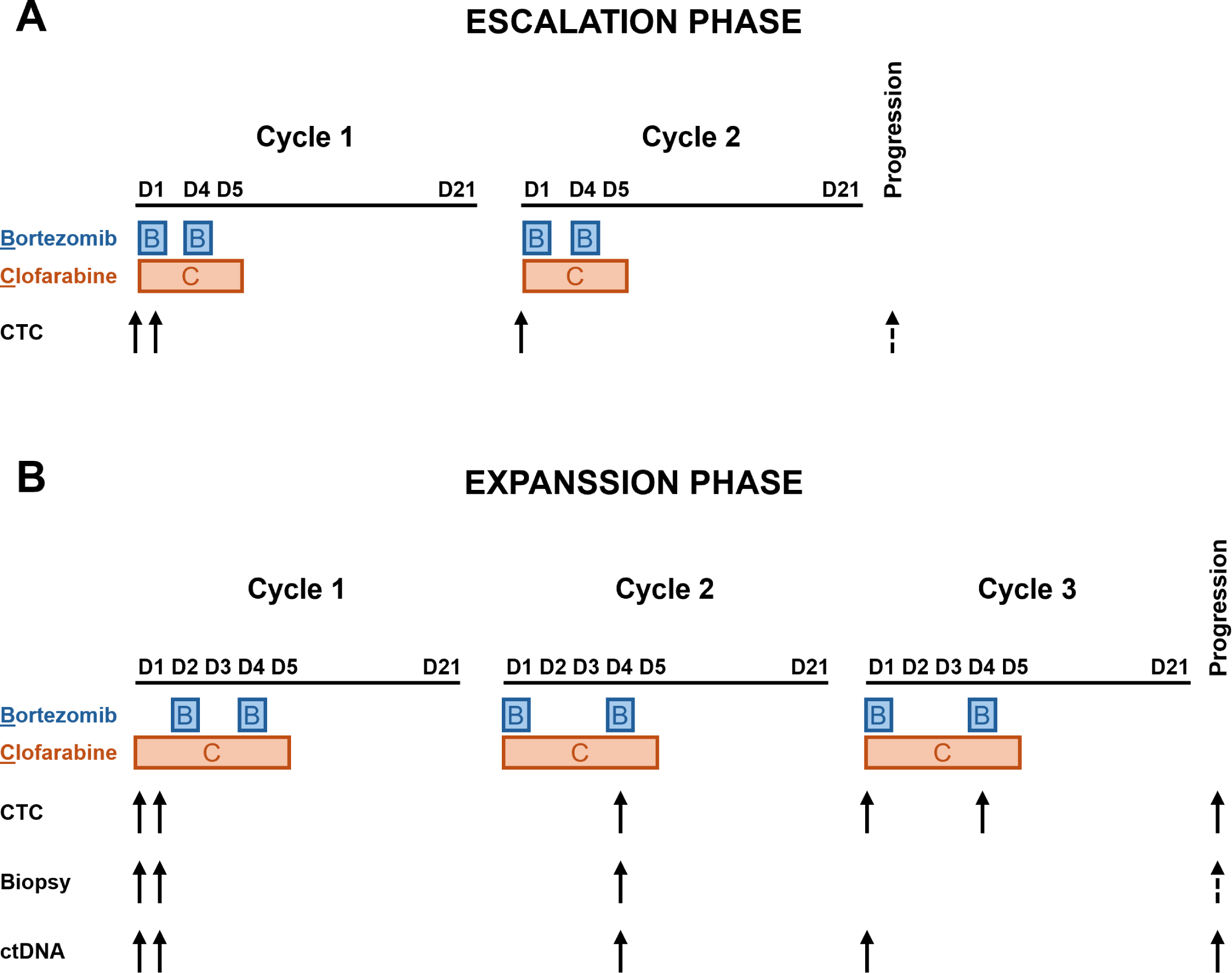

Supplement: Supplementary file 1 — Supplementary Fig. 1: Study Schema for The Phase 1 Clinical Trial. (A) Dose-escalation cohort: Bortezomib was administered subcutaneously on days 1 and 4 and clofarabine was administered intravenously days 1–5 of each 21-day cycle. Mandatory blood samples for circulating tumor cell (CTC) analysis were collected from patients with solid tumors only at baseline (pre-treatment), on cycle 1 day 2 prior to clofarabine administration and on day 1 of all subsequent cycles before drug administration. Optional blood samples for CTC analysis could also be collected at time of progression. (B) Dose-expansion cohort: Bortezomib was administered subcutaneously on days 2 and 4 and clofarabine was administered intravenously days 1–5 of each 21-day cycle. Mandatory biopsies were collected prior to clofarabine and bortezomib administration (up to 8 days prior to the start of treatment), on cycle 1 day 1 (2–4 h post-clofarabine), and cycle 2 day 4 or 5; an optional biopsy could be collected at time of disease progression. Blood samples for CTCs analysis were collected at baseline (pre-treatment); on cycle 1 day 1 (2–5 h after clofarabine administration); at the time of the biopsy on cycle 2 day 4 or day 5 (within ± 8 h of the biopsy); on days 1 and 4 of cycle 3 and all subsequent cycles before drug administration; and at time of disease progression. Blood samples for circulating tumor DNA (ctDNA) analysis were collected at baseline (pre-treatment); at the time of the cycle 2 (day 4 or 5) biopsy; on day 1 of cycle 3 and all subsequent cycles; and at time of disease progression. Full arrows indicate mandatory planned sample collections; dotted arrows indicate optional sample collections [file 280_2026_4868_MOESM1_ESM.tif]
